# Assessment of Central Visual Function in Patients with Retinitis Pigmentosa

**DOI:** 10.1038/s41598-018-26231-9

**Published:** 2018-05-23

**Authors:** Kohta Fujiwara, Yasuhiro Ikeda, Yusuke Murakami, Takashi Tachibana, Jun Funatsu, Yoshito Koyanagi, Shunji Nakatake, Noriko Yoshida, Shintaro Nakao, Toshio Hisatomi, Shigeo Yoshida, Takeshi Yoshitomi, Tatsuro Ishibashi, Koh-Hei Sonoda

**Affiliations:** 1 Department of Ophthalmology, Graduate School of Medical Sciences, Kyushu University, Fukuoka, 812-8582 Japan; 2 Department of Ophthalmology, Graduate School of Medical Sciences, Akita University, Akita, 010-8543 Japan

## Abstract

In order to clarify the disease progression in retinitis pigmentosa (RP) and its related factors, reliable data on the changes in central visual function in RP are needed. In this longitudinal study, we examined 118 patients who were diagnosed with typical RP. Visual acuity (VA), visual field using a Humphrey Field Analyzer with the central 10-2 SITA-Standard program, and optical coherence tomography measurements were obtained. The slopes, which were derived from serial values of mean deviation (MD), macular sensitivity (MS), or foveal sensitivity (FS) obtained for each eye by a linear mixed model, were used for analysis. MS and FS were calculated as the average retinal sensitivity of 12 and 4 central points respectively. There were statistically significant interactions of times with levels of the central subfield thickness (CST) on the slopes of MS and FS. Compared to the eyes without macular complications, the eyes with macular complications had steeper MD, MS and FS slopes, and this interaction was no significant, but marginal trend for the MS or FS slope (P = 0.10, 0.05, respectively). The central retinal sensitivity (i.e., MS and FS) slopes calculated were effective indices of the progression of central visual function in RP.

## Introduction

Retinitis pigmentosa (RP) is a group of inherited retinal degeneration diseases resulting from photoreceptor cell death, and individuals with RP typically suffer from impaired night vision and a gradual loss of the visual field^1^. Unfortunately, no effective treatment exists for RP at present, but some promising therapeutic treatments have been suggested by animal studies^2–4^. As a next step, the efficacy of these treatments should be assessed in clinical trials; however, appropriate parameters for the assessment of disease progression that could be used in therapeutic clinical trials for RP patients have not yet been established.

In the present study, we focused on central visual function, which involves primarily cone photoreceptor cells^5^. We previously reported that macular sensitivity (MS) calculated as the average retinal sensitivity of 12 central points on the HFA 10-2 program was increased concomitant with a reduction of central macular thickness measured by optical coherence tomography (OCT) in RP patients with cystoid macular edema (CME)^6,7^, indicating that the MS is a valid parameter for use in evaluating central visual function. Moreover, Iijima demonstrated that not only mean deviation (MD) values but also the average sensitivity of the central 12 points (MS) and that of the central four points (we refer to this as “foveal sensitivity” [FS] herein) on the HFA10-2 program were correlated with the visual acuity (VA) converted into logMAR, especially in advanced RP^8^. Thus, it is possible that MS and FS would be useful parameters of central visual function in patients with RP. However, there have been no studies assessing longitudinal central visual function and its related factors.

RP is also a chronic progressive disease, and it is thus important to be able to measure the slight changes of central visual function^9^, which could be affected by macular complications. A method for the long-term assessment of central visual function in RP patients is needed. VA is easy to measure, whereas it is difficult to identify the slight changes in RP. Goldmann visual field testing has been used as a general and standard perimetry method for patients with RP^10–12^, but it is difficult to quantify the central progression of RP as represented by numerical values. Static perimetry using a Humphrey Field Analyzer (HFA) and Octopus^TM^ perimeter has been conducted to detect visual field loss^13,14^. Hirakawa *et al*. investigated the progression of defects in the central 10-degree visual field in patients with RP and choroideremia using the HFA 10-2 program, and found that the MD value obtained using the HFA 10-2 program was a suitable parameter for monitoring and demonstrating disease progression in RP^15^. Birch *et al*. showed that dark-adapted visual fields were obtained on a modified Octopus^TM^ 201 automated perimeter. They studied the relationships between the rod visual field area using dark-adapted visual fields and the parameters derived from full-field rod ERG^13^.

In the present study, we studied the decline of central visual function in RP patients using the HFA 10-2 program. We also investigated the associations among several factors related to RP disease progression.

## Results

The demographic data of the study subjects are summarized in Table 1. Sixty-four percent of the patients were female, the mean age of the total group was 47.1 years old, and the mean follow-up was 5.3 years. Table 1 also shows the mean values or frequencies of visual factors in the total of 118 patients with RP.

**Table 1:** Characteristics of the 118 patients with retinitis pigmentosa.

Variable	All	Inheritance mode		
		AD	AR + Sporadic	P Value
Patients, n	118	20	98	
Sex, female (%)	77 (65)	15 (75)	62 (63)	0.46
Follow-up, yrs	5.3 (1.8–8.8)	5.3 (1.8–8.0)	5.4 (1.8–8.8)	0.36
HFA measurements, times	8.6 ± 4	7.6 ± 3	8.8 ± 4	0.19
Parameters at baseline				
Age, yrs	47.1 ± 15	42.1 ± 15	48.2 ± 15	0.11
CST, μm	272 ± 80	274 ± 95	271 ± 77	0.88
VA, Log MAR	0.11 ± 0.2	0.06 ± 0.1	0.13 ± 0.2	0.31*
MD, dB	−12.3 ± 9.4	−12.4 ± 8.7	−12.2 ± 9.6	0.93
MS, dB	28.4 ± 7.2	28.7 ± 7.3	28.3 ± 7.2	0.84
FS, dB	30.2 ± 6.0	30.9 ± 5.6	30.0 ± 6.1	0.58

Values are given as the mean ± standard deviation or as percentages. AD, autosomal dominant retinitis pigmentosa (RP); AR, autosomal recessive RP; HFA, Humphrey Field Analyzer; FS, foveal sensitivity; CST, central subfield thickness; VA, visual acuity; MD, mean deviation; MS, macular sensitivity.

*Mann-Whitney test.

**Figure 1:**
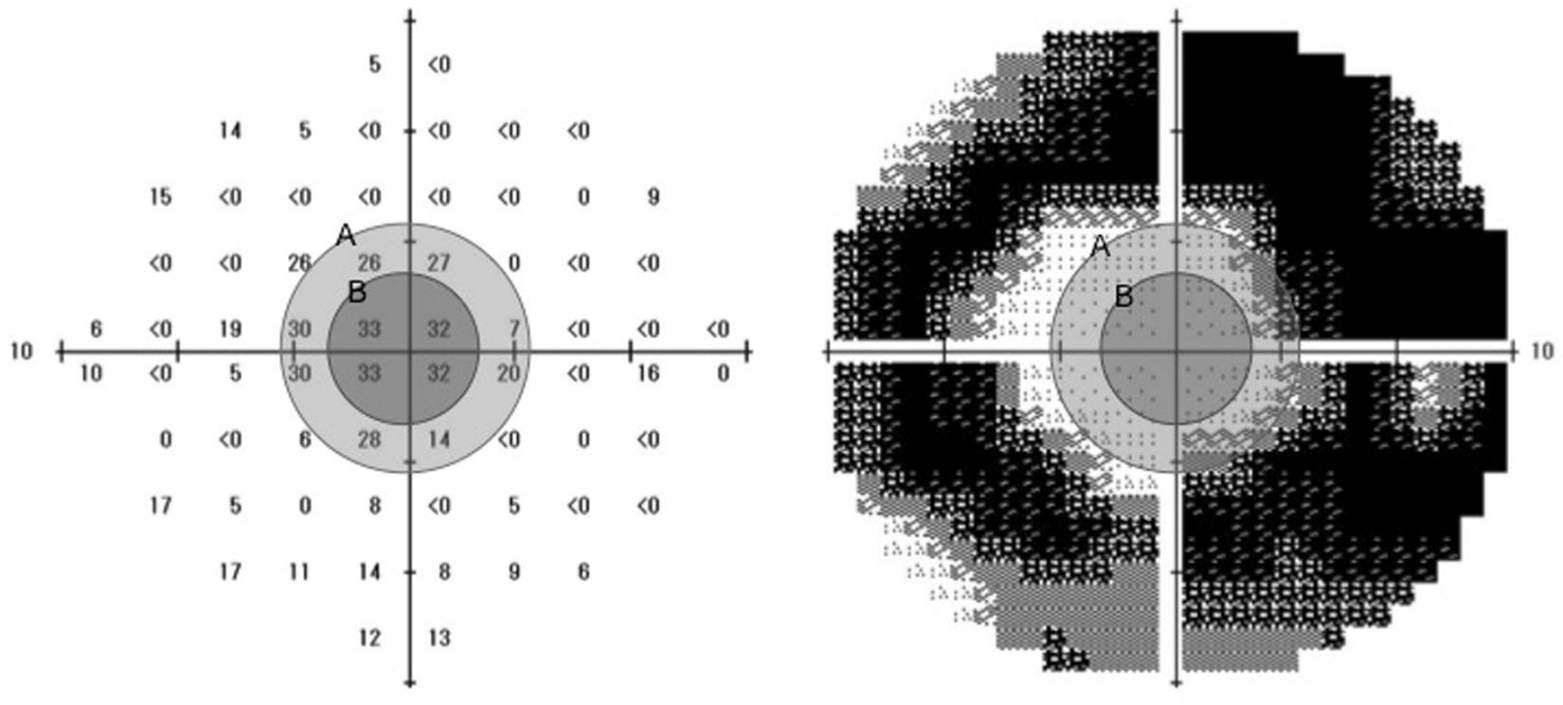
The central 10-2 SITA-Standard program. The macular sensitivity was calculated as the average of the 12 central points (circle A). The foveal sensitivity was also calculated as the average of the 4 central points (circle B).

**Figure 2:**
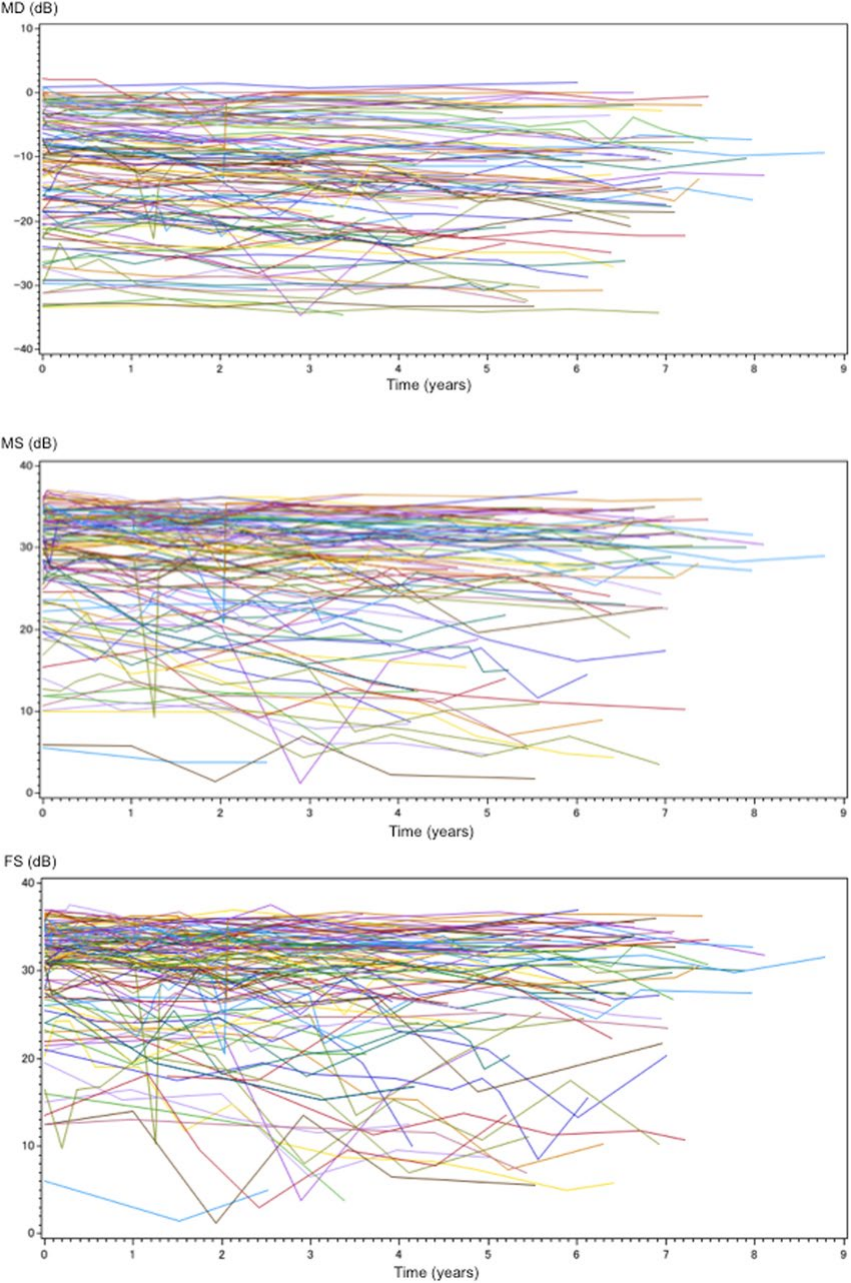
Plots of the visual sensitivities of the follow-up years in 118 patients. The mean deviations of all sections are shown in the top row, the macular sensitivity in the second row, and the foveal sensitivity in the third row.

The MS and FS calculated as the average of the 12 and 4 central points (excluding foveal points) are shown in Fig. 1. The MD and sensitivity for MS and FS at each follow-up time are plotted for each patient in Fig. 2. Table 2 shows the calculated mean reduction of these parameters as the coefficients in the group of all RP patients.

**Table 2:** Visual field progression of retinitis pigmentosa patients.

Variable	Coefficient	95% CI	P value
MD, dB	−0.47	−0.58 to −0.37	<0.0001
MS, dB	−0.58	−0.73 to −0.43	<0.0001
FS, dB	−0.55	−0.69 to −0.40	<0.0001

Average progression rate of MD, MS, and FS using

linear mixed models. Random intercepts and random

slopes for each subject are adopted for analysis.

The coefficient of time (years) of each model is the fixed

effect of the slopes, or the average progression rate.

CI, confidence interval.

We next estimated the coefficients and 95% confidence intervals for the decline of sensitivity by times (years) according to the levels of several confounding factors—namely, sex, age, mode of inheritance (autosomal dominant or not) and the central subfield thickness (CST) (Table 3). The MS and FS slopes differed significantly between eyes with different CST levels (P = 0.01, P = 0.001, respectively).

**Table 3:** Visual field progression of retinitis pigmentosa patients in the subgroups of variables.

Variable	n	Coefficient	95% CI	P value	P for interaction
MD					
Sex (men)	41	−0.47	−0.69 to −0.26	<0.0001	0.12
Sex (women)	77	−0.48	−0.60 to −0.35	<0.0001	
MS					
Sex (men)	41	−0.64	−0.94 to −0.34	<0.0001	0.66
Sex (women)	77	−0.56	−0.74 to −0.38	<0.0001	
FS					
Sex (men)	41	−0.62	−0.92 to −0.33	<0.0001	0.53
Sex (women)	77	−0.52	−0.69 to −0.34	<0.0001	
MD					
Age, ≤ 46 (years)	57	−0.49	−0.63 to −0.35	<0.0001	0.72
Age, > 46 (years)	61	−0.45	−0.60 to −0.30	<0.0001	
MS					
Age, ≤ 46 (years)	57	−0.48	−0.67 to −0.29	<0.0001	0.18
Age, > 46 (years)	61	−0.68	−0.92 to −0.45	<0.0001	
FS					
Age, ≤ 46 (years)	57	−0.47	−0.67 to −0.26	<0.0001	0.32
Age, > 46 (years)	61	−0.62	−0.83 to −0.40	<0.0001	
MD					
AD	20	−0.44	−0.69 to −0.18	0.002	0.75
AR + Sporadic	98	−0.52	−0.93 to −0.10	<0.0001	
MS					
AD	20	−0.64	−0.94 to −0.35	0.0002	0.73
AR + Sporadic	98	−0.57	−0.74 to −0.40	<0.0001	
FS					
AD	20	−0.66	−0.95 to −0.37	0.0001	0.51
AR + Sporadic	98	−0.52	−0.69 to −0.36	<0.0001	
MD					
CST, > 252	59	−0.47	−0.62 to −0.32	<0.0001	0.81
CST, ≤ 252	59	−0.49	−0.64 to −0.34	<0.0001	
MS					
CST, > 252	59	−0.39	−0.53 to −0.25	<0.0001	0.01
CST, ≤ 252	59	−0.78	−1.04 to −0.52	<0.0001	
FS					
CST, > 252	59	−0.30	−0.43 to −0.17	<0.0001	0.001
CST, ≤ 252	59	−0.79	−1.04 to −0.54	<0.0001	

Average progression rate of MD, MS, and FS using linear mixed models in the subgroups of variables. The patients were divided into two groups by median baseline age and CST. Random intercepts and random slopes for each subject are adopted for analysis. The coefficient of time (years) of each model is the fixed effect of the slopes, or the average progression rate.

CI, confidence interval.

To avoid the effects of therapeutic treatments, we excluded the patients who had undergone treatment with topical unoprostone isopropyl for neuroprotection^16^ or topical dorzolamide (a carbonic anhydrase inhibitor) for CME^6,7^ in the following analysis. We determined the mean values of visual field progression for the eyes without macular complications (n = 46) and those with macular complications (n = 16) (Table 4). Compared to the eyes without macular complications, those with macular complications had steeper MD, MS and FS slopes, and this interaction was no significant, but marginal trend for the MS or FS slope (P = 0.10, 0.05, respectively). Moreover, in each of the 34 patients with no macular complications, there was no significant interaction in the progression of central visual function between the right and left eyes (Table 5).

**Table 4:** Visual field progression of retinitis pigmentosa patients by status of macular complication.

Variable	n	Coefficient	95% CI	P value	P for interaction
MD					
Macular complication (−)	44	−0.46	−0.66 to −0.26	<0.0001	0.30
Macular complication (+)	16	−0.65	−1.02 to −0.29	0.002	
MS					
Macular complication (−)	44	−0.50	−0.81 to −0.19	0.002	0.10
Macular complication (+)	16	−0.98	−1.51 to −0.46	0.001	
FS					
Macular complication (−)	44	−0.49	−0.80 to −0.19	0.002	0.05
Macular complication (+)	16	−1.08	−1.68 to −0.49	0.002	

Average progression rate of MD, MS, and FS using linear mixed models in the subgroups of

variables. The patients were divided into two groups by the presence or absence of macular

complication. Random intercepts and random slopes for each subject are adopted for

analysis. The coefficient of time (years) of each model is the fixed effect of the slopes, or

the average progression rate.

CI, confidence interval.

**Table 5:** Visual field progression of right and left eyes with retinitis pigmentosa.

Variable	n	Coefficient	95% CI	P value	P for interaction
MD					
Right eye	34	−0.47	−0.71 to −0.23	0.0004	0.56
Left eye	34	−0.59	−0.87 to −0.31	0.0001	
MS					
Right eye	34	−0.47	−0.87 to −0.07	0.02	0.33
Left eye	34	−0.56	−0.94 to −0.18	0.005	
FS					
Right eye	34	−0.48	−0.88 to −0.09	0.02	0.35
Left eye	34	−0.59	−1.07 to −0.11	0.02	

Average progression rate of MD, MS, and FS using linear mixed models. Random intercepts and random slopes for each subject are adopted for analysis. The coefficient of time (years) of each model is the fixed effect of the slopes, or the average progression rate.

CI, confidence interval.

## Discussion

We defined the progression rate of visual field loss and elucidated the factors that contribute to this progression rate in patients with RP, using automated static perimetry (the HFA 10-2 program). To our knowledge, this is the first study to define the progression of central visual function based on the long-term follow-up data in over 100 RP patients. The key findings obtained in our study were that (1) the slopes in the plot of the average of central retinal sensitivity (i.e., MS and FS) calculated by the HFA 10-2 program represented the progression of central visual function in RP patients, (2) CST measured by OCT, were associated with disease progression, and (3) in each of the 34 patients with no macular complications, there was no significant difference in the progression of central visual function between the right and left eyes.

In studies evaluating the therapeutic efficacy of various treatments for patients with RP, cone ERG amplitude measurement (30-Hz flicker ERGs)^17^, microperimetry^18,19^, and the HFA 10-2 program^20,21^ were used. However, there are no standard criteria for the assessment of the progression of central visual function in RP, because RP is a rare and chronic progressive disease. To evaluate the efficacy of promising treatments in clinical trials, we must identify the parameters that accurately represent disease progression.

As noted above in the Introduction, Hirakawa *et al*. showed that the MD value obtained with the HFA 10-2 program is a suitable parameter representing disease progression in RP^20^. Regarding the “quality of vision,” central visual function is important for individuals with RP, and the change of MD value is affected mainly by peripheral vision loss rather than central visual function in individuals with RP. Our clinical studies demonstrated MS improvement following a reduction of the CME^6,7^. In the present study, we observed no significant, but marginal trend for the MS or FS slope (P = 0.10, 0.05, respectively) between the eyes with and without macular complications (Table 4). Thus, the parameters of the average of central retinal sensitivity (i.e., MS and FS) measured by the HFA 10-2 program are expected to reflect central visual function with good precision.

RP is frequently associated with common complications such as cataract^22–25^, CME^6,7,15,26^, and ERM^27–29^. We speculated that the progression of visual function in RP is affected by both retinal degeneration and these complications. Therefore, in the first analysis (Table 2), we also included patients with any macular complication. For the development of new and more effective treatments for rare diseases such as RP, analyses of all of the historical data are useful for the assessment of efficacy, and we thus considered the progression of visual function without macular complications as the historical control for future clinical trials, as shown in Table 4. Moreover, our present investigation demonstrated that there are no significant differences in the changes of visual function (i.e., in either the MD, MS, or FS slopes) between the right and left eyes of each of 34 patients (Table 5), suggesting that the fellow eye can be used as a control for therapeutic evaluations in one eye.

Lupo *et al*. reported a correlation between retinal thickness and visual function in RP^30^. Lenassi *et al*. also revealed a high correlation between retinal sensitivity and outer retinal thickness values measured by both static and kinetic fundus-related perimetry with structural changes in the inner and outer retina evaluated with SD-OCT^31^. Our present study showed that there is a significant interaction in the association between CST and central visual function (Table 3). This association indicates that the CST could represent both the current central visual function at the moment and the further progression of macular dysfunction.

Our present findings are significant because of the study’s large sample size and long follow-up time. Nevertheless, there are some limitations that should be discussed. First, we performed the calculation of threshold sensitivities according to the previous investigations, since using threshold sensitivities is a common method and generally acceptable at this time. However, It may be unsuitable to average the sensitivities (dB) of each point because the dB is logarithmic scale. When calculating MS and FS, Sayo converted dB to 1/Lambert, and then average the values, not simply to average the sensitivities (dB)^32^. In future study, this method seem to be standard for measurement of retinal sensitivities. Moreover, further studies are needed to compare the both macular sensitivities calculated by threshold sensitivities or based upon TD plot values, as Sayo described^32^. Second, RP is caused by mutations in more than 50 responsible genes^33^, and RP patient series therefore present a genetically heterogeneous group of retinal degenerative diseases. However, we evaluated the progression of visual function without considering this heterogeneity in this patient series. Third, since the distribution of inherited modes differs among ethnic groups^34^, in our hospital-based study comprised of Asian peoples, there were few X-linked RP patients, and thus we could not demonstrate the development of X-linked RP patients, who have a steeper decline of visual field loss than other inherited groups. Taking the above into account, we are now attempting to create an RP patient registry with pathogenic genetic information, and we plan to assess the progression of visual function for each genetic mutation in a future study.

In conclusion, the slopes in the average of central retinal sensitivity (i.e., MS and FS) calculated by the HFA 10-2 program are effective for representing the progression of central visual function in RP.

## Methods

### Study Design and Ethics Statement

We retrospectively studied the records of patients with RP and obtained examination results including HFA measurements, spectral domain OCT (SD-OCT) findings (Cirrus HD-OCT, Carl-Zeiss Meditec, Dublin, CA), and visual and systemic parameters.

This study was approved by the Institutional Review Board of Kyushu University Hospital (Fukuoka, Japan) and was conducted in accord with the tenets of the Declaration of Helsinki on Biomedical Research Involving Human Subjects. The review board waived the need for written informed consent because the study design was a retrospective chart review.

### Patients

Patients were enrolled at Kyushu University Hospital from 2008 to 2014: 225 patients with a diagnosis of typical RP underwent automated static perimetry (HFA) testing. Patients who consecutively underwent at least three HFA examinations were included in the present study. The HFA measurement intervals were 3 to 12 months irregularly. The eyes of patients who had less than three HFA examinations and those who had a history of other ocular diseases or intraocular surgery (e.g., cataract surgery) during follow up were excluded. After this exclusion, a total of 118 of the original 225 patients were enrolled. The examination results of the right eye of each subject were used for analysis. In cases in which the right eye could not be examined due to exclusion criteria, we used the left eye for analysis. The best-corrected VA was measured with a standard Japanese decimal VA chart and was converted to the logarithm of the minimal angle of resolution (logMAR) units.

The diagnosis of typical RP was based on a history of night blindness, visual field constriction and/or ring scotoma, and markedly reduced or non-recordable a- and b-wave amplitudes on electroretinography testing, in addition to ophthalmoscopic findings (e.g., bone spicule-like pigment clumping in the mid-peripheral and peripheral retina and attenuation of retinal vessels). Sporadic cases were grouped together with the autosomal recessive (AR) cases, because most of the mutations of sporadic cases are thought to be recessive.

### Visual Field Testing

All patients underwent automated static perimetry with the central 10-2 SITA-Standard program. The lens was corrected as appropriate for the test distance. Visual field testing was repeated if the test reliability was not satisfactory (i.e., fixation loss > 20%, false positive > 15%, or false negative > 33%). The test was conducted twice, and the better result was used for the analysis in order to reduce the learning effect. The calculation of the MD value involved averaging the differences between the measured sensitivities and the age-adjusted normal sensitivities (total deviations) at each test point. The MS was calculated as the average of the 12 central points (excluding foveal points) as described previously (Fig. 1)^6,7^. We also calculated the FS as the average of the four central points, excluding foveal points.

The linear mixed model has been well recognized as an effective method for obtaining estimates of individual reductions in vision^32,35^. For analysis of the parameters of MD, MS and FS, we used the serial values of MD, MS, or FS obtained for each eye by linear mixed model as the MD slope, MS slope and FS slope, respectively. Random intercepts and random slopes for each subject are adopted for analysis. Coefficient of time (years) of each model is the fixed effect of the slopes, or the average progression rate. Moreover, the average progression rate of MD, MS, and FS was calculated using linear mixed models in the subgroups of variables. The patients were divided into two groups by median of baseline age and CST. If reliable data were not obtained in repeated tests, the data were not included in the study.

### Definition of Macular Complications

OCT images taken on the same day of the HFA baseline measurements were analyzed. Research software (Carl Zeiss Meditec) was used to measure the CST. The average thickness of all points within the inner 1-mm diameter circle was defined as the CST of the fovea. The epiretinal membrane (ERM), CME and macular hole (MH) were detected by fundus examination and by SD-OCT. The diagnosis of ERM using SD-OCT is based on the presence of a hyper-reflective line or band over the retinal surface, frequently associated with wavy changes in the underlying retina^29,36^.

### Statistical Analysis

The data are presented as the mean ± standard deviation (SD). Mean values were compared using Student’s *t*-test and Mann-Whitney test, and differences in frequencies were compared using the chi-square test. The linear mixed models were used to examine the slopes and interactions with sex, age, mode of inheritance (autosomal dominant or not), CST, and the macular complications. All of the statistical analyses were performed with SAS software, ver. 9.3 (SAS Institute, Cary, NC). Two-sided p-values < 0.05 were considered significant.
